# Specialized Nutritious Food Combined With Cash Transfers and Social and Behavior Change Communication to Prevent Stunting Among Children Aged 6 to 23 Months in Pakistan: Protocol for a Cluster Randomized Controlled Trial

**DOI:** 10.2196/19001

**Published:** 2020-08-24

**Authors:** Gul Nawaz Khan, Sumra Kureishy, Shabina Ariff, Muhammad Atif Habib, Asra Abeer Usmani, Areeba Mubarik, Masawar Hussain, Naveed Akbar, Pablo Rodriguez de Castro, Alba Cecilia Garzon, Saskia de Pee, Sajid Bashir Soofi

**Affiliations:** 1 Department of Paediatrics and Child Health Aga Khan University Karachi Pakistan; 2 World Food Programme Islamabad Pakistan; 3 Medical College Aga Khan University Karachi Pakistan; 4 Benazir Income Support Programme Government of Pakistan Islamabad Pakistan; 5 World Food Programme Rome Italy

**Keywords:** stunting, cash transfers, specialized nutritious food, social and behavior change communication, Pakistan

## Abstract

**Background:**

In Pakistan, the prevalence of stunting in children younger than 5 years has remained above global critical levels over the past two decades, with the stunting rate being 40.2% in 2018. Children living in rural areas and in the poorest households suffer the most from stunting across the country—43.2% in rural areas and 51.4% in the lowest wealth quintile. As a continuing public health concern, it is essential that stunting prevention is a national priority in order to ensure human capital development, especially among the poorest households.

**Objective:**

The primary objective of this study is to determine the effect of a medium quantity of a lipid-based nutrient supplement (LNS) combined with unconditional cash transfers and social and behavior change communication (SBCC) on reduction of stunting in children aged 6 to 23 months.

**Methods:**

A 5-arm cluster randomized controlled trial will be conducted in the district of Rahim Yar Khan in Punjab, Pakistan. The intervention packages will be (1) cash only, (2) cash with LNS, (3) cash with SBCC, and (4) cash with SBCC and LNS. The control arm will receive routine standard of care. We will enroll children at 6 months of age and follow up on a monthly basis up to 24 months of age. A total of 2000 children, 400 in each arm, will be enrolled to detect a 20% reduction in the prevalence of stunting among children aged 24 months. Length, weight, food intake, compliance to interventions, morbidities, and other relevant data will be collected at enrollment and on a monthly basis over the period of 18 months. The process evaluation will assess acceptability of the interventions and potential barriers to implementation through focus group discussions and in-depth interviews with the target population and relevant stakeholders. Furthermore, a cost analysis will be conducted to assess the cost-effectiveness of each intervention package.

**Results:**

The study protocol was approved by the Ethics Review Committee of Aga Khan University in Pakistan on January 4, 2017. Data collection began in May 2017 and was completed in July 2019. Data analyses are yet to be completed. This study will explore the effectiveness of intervention packages comprised of cash transfers from Benazir Income Support Programme with or without additional LNS and SBCC in preventing childhood stunting. We expect the results to be published in peer-reviewed journals by autumn of 2020.

**Conclusions:**

The findings of this trial will provide robust evidence as to which intervention packages can have significant effects on linear growth of children and design effective intervention packages to prevent stunting in children aged 6 to 23 months.

**Trial Registration:**

ClinicalTrials.gov NCT03299218; https://clinicaltrials.gov/ct2/show/NCT03299218

**International Registered Report Identifier (IRRID):**

DERR1-10.2196/19001

## Introduction

Child stunting remains a major barrier to human capital development worldwide. In 2018, a total of 149 million, or 22%, of children younger than 5 years worldwide were found to be stunted [1]. South Asia has 57.9 million stunted children younger than 5 years, one of the highest burdens of stunting across regions [2].

In Pakistan, the prevalence of stunting in children younger than 5 years has remained above global critical levels over the last two decades, with the stunting rate being 40.2% in 2018. Children living in rural areas and in the poorest households suffer the most from stunting across the country—43.2% in rural areas and 51.4% in the lowest wealth quintile [3].

Stunting has underlying factors that expand beyond nutrition, health, and agriculture to include poverty and social vulnerability. Social protection is increasingly recognized globally as a strategic tool to improve maternal and child nutrition outcomes due to its ability to alleviate poverty and social vulnerability. In particular, cash transfers have become increasingly popular as an effective, efficient, and welcome means for welfare improvement of low-income households in many low- and middle-income countries (LMICs) [4-6]. Cash transfers may be in the form of unconditional cash transfers (UCTs) or conditional cash transfers, which are provided on the condition that recipient households must carry out certain actions (such as uptake of health, education, or nutrition services) [7].

A conditional cash transfers study from Mexico reported a significant positive impact on child growth, with an increase of 16% in the mean child growth per year (1 cm per year), and a positive effect on the consumption of vitamins and minerals among beneficiary households [8]. A review on the impact of conditional cash transfers in LMICs concluded that cash transfers had a positive impact on nutritional status [9]. Estimates of cash transfer programs in Africa indicated a positive effect on anthropometric status [10-12].

Evidence shows that people can change their behaviors to improve nutrition outcomes when provided with financial support to afford nutritious foods [13]. Through the Maternal and Child Cash Transfers for Improved Nutrition program in Myanmar, pregnant women in targeted villages were provided with monthly cash transfers of K 15,000 (US $10.50) and social and behavioral change communication (SBCC) until their child was aged 2 years [14,15]. The cash transfer aimed to support women’s ability to afford nutritious food for themselves and their children. A recent impact evaluation study of the program revealed that cash transfers combined with SBCC reduced stunting by 4 percentage points and wasting by 3 percentage points compared with only cash transfers. However, with the limited size of the cash transfers (US $10-15 per month) and the large affordability gap experienced in the poorest 20% to 30% of households, in-kind nutritional supplementation is essential in order to prioritize improving nutrition for the most vulnerable, pregnant and lactating women (PLW) and children younger than 2 years [14,15].

Lipid-based nutrient supplements (LNS) were developed for the treatment of moderate acute malnutrition and prevention of undernutrition among children aged 6 to 59 months. [16]. Many intervention studies showed contrasted effects on the incidence of wasting and stunting and on mean change in weight for height, weight gain, height for age, and height gain, with some experiments demonstrating positive results and others indicating no impact [17-25]. The variations in outcomes of these studies might be due to differences across studies, such as targeting of children of various age groups and nutritional statuses, use of different types and quantities of supplements, different study designs, and implementation in different contexts.

Punjab, where half of the population of Pakistan lives, has one of the highest burdens of stunting (36.4%). Nearly 47% of the children from the poorest households in the province are stunted. Based on provincial statistics, the highest rate of stunting and poverty can be seen in Southern Punjab, especially in the district of Rahim Yar Khan, where more than 20% of the people live in extreme poverty and 2 out of 5 children younger than 5 years are stunted. Among the poorest 20% of households, 1 out of 2 children younger than 5 years are stunted in the district of Rahim Yar Khan in Punjab [3].

The Aga Khan University (AKU) will conduct a cluster randomized controlled trial in the district of Rahim Yar Khan in collaboration with the Integrated Reproductive Maternal Newborn, Child Health & Nutrition Program and the Benazir Income Support Programme (BISP). The intervention packages include LNS, UCTs, and SBCC, targeting the current BISP beneficiary households (poorest 20% of households) of the district. The World Food Programme provided the funds.

The primary objective of this study is to determine the effect of the unconditional cash transfer program alone and combined with behavior change communication, specialized nutritious food supplementation, or both on reduction in the prevalence of stunting among children aged 24 months. Secondary objectives include reduction in the prevalence of wasting and underweight, improvement in the micronutrient status and infant and young child feeding practices, and evaluation of the cost-effectiveness of different packages for reduction of stunting among children aged 24 months.

## Methods

### Study Setting

The study will be conducted in the district Rahim Yar Khan in Punjab, Pakistan. The district is located in the southern part of Punjab province, with an area of 11,880 square kilometers. The district is administratively subdivided into 4 tehsils and 122 union councils and has a population of 4.8 million (79% living in rural areas). According to the 2018 Multiple Indicators Cluster Survey conducted in Punjab, the literacy rate is 43.1% among women and 58.1% among men aged 15 to 49 years in Rahim Yar Khan [26]. Within the district, 13.1% of the population has access to improved sources of drinking water (piped water), 75.6% have access to improved sanitation, and 90.6% have access to electricity. As a predominately agrarian district, only 7.8% and 56.2% of households own agriculture land and livestock, respectively. Furthermore, infant mortality (56 infant deaths per 1000 live births) and under-5 mortality (66 child deaths per 1000 live births) are similar to the provincial (60 infant deaths per 1000 live births and 69 child deaths per 1000 live births) and national averages (62 infant deaths per 1000 live births and 74 child deaths per 1000 live births) [26].

### Interventions

A combination of preventative interventions will be implemented to address stunting among children aged 6 to 23 months from BISP beneficiary households. The interventions will consist of locally produced LNS for children aged 6 to 23 months, a UCT delivered through BISP for households with children aged 6 to 23 months, and SBCC focused on health, nutrition, and hygiene delivered through lady health workers (LHWs) (Textbox 1). The nutritional value of the LNS is shown in Table 1. The control group will receive routine public health services available within the area.

**Intervention packages.**

**Unconditional cash transfers**

A total of Rs 5000 (US $32) on a quarterly basis will be transferred by the Benazir Income Support Programme throughout the study period. Participants will be able to collect their cash transfers from automated teller machines in the form of direct cash after biometric verification.

**Lipid-based nutrient supplement – medium quantity**

A locally produced lipid-based nutrient supplement (called Wawamum) will be provided to children aged 6 to 23 months by the study team. Wawamum is made with heat-treated (roasted) chickpeas, vegetable oils, dry skim milk, sugar, vitamins, minerals, emulsifier, and antioxidants (Table 1). A daily ration of 50 grams of Wawamum (1 sachet) will be provided to cover the recommended daily allowance of most micronutrients and 260 kilocalories of energy (about a quarter of daily energy requirements). Each recruited child will receive Wawamum for a duration of 18 months from 6 months to 24 months of age.

**Social and behavior change communication (SBCC)**

SBCC messages will be delivered by the lady health workers from the existing public health system during their routine monthly house-to-house visits and in community sessions on a quarterly basis with the help of a specialized picture booklet. Lady health workers will receive 2 days of intensive SBCC training before the initiation of the study enrollment and a 1-day refresher training annually, with special emphasis on communication skills, introduction of complementary feeding, and dietary diversity. All enrolled mothers will receive 18 individual house-to-house visits and 6 community sessions. Other household and community members will also be encouraged to participate in the sessions.

**Table 1:** Nutritional values of lipid-based nutrient supplement—medium quantity.

Nutritional values (per 50 g/1 serving)	Minimum	Maximum
Energy, kcal	255	280
Protein, g	5.5	8
Fat, g	13	18
ω-3 fatty acids, g	0.15	0.9
ω-6 fatty acid, g	1.3	3.1
Retinol (Vitamin A), μg	275	575
Thiamin (Vitamin B_1_), mg	0.5	—^a^
Riboflavin (Vitamin B_2_), mg	1.05	—
Niacin (Vitamin B_3_), mg	6.5	—
Pantothenic Acid (Vitamin B_5_), mg	2	—
Pyridoxine (Vitamin B_6_), mg	0.9	—
Biotin (Vitamin B_7_), μg	30	—
Folate (Vitamin B_9_), μg, DFE^b^	165	—
Cyanocobalamin (Vitamin B_12_), μg	1	—
Ascorbate (Vitamin C), mg	30	—
Cholecalciferol (Vitamin D), μg	7.5	10
Tocopherol Acetate (Vitamin E), mg, ATE^c^	8	—
Phytonadione (Vitamin K), μg	13.5	—
Calcium, mg	268	375
Copper, mg	0.7	1.0
Iodine, μg	50	70
Iron, mg	5	7
Magnesium, mg	75	113
Manganese, mg	0.6	1.2
Phosphorus, mg	225	375
Potassium, mg	450	700
Selenium, μg	10	20
Sodium, mg	—	135
Zinc, mg	5.5	7

^a^Not available.

^b^DFE: dietary folate equivalents.

^c^ATE: α-tocopherol equivalents.

### Study Design

**Figure 1:**
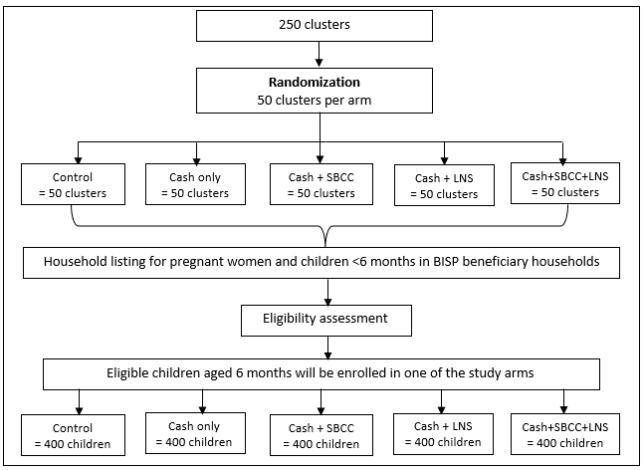
CONSORT flow diagram of the study. BISP: Benazir Income Support Programme; CONSORT: Consolidated Standards of Reporting Trials; LNS: lipid-based nutrient supplement; SBCC: social and behavior change communication.

A 5-arm cluster randomized controlled trial will be conducted to examine the effectiveness and cost-effectiveness of UCT, LNS, SBCC, and their combinations to prevent stunting in children aged 6 to 23 months living in BISP beneficiary households. The 5 study arms will be (1) control group, which will receive routine government health services; (2) BISP cash transfers; (3) BISP cash transfers and SBCC; (4) BISP cash transfers and LNS; and (5) BISP cash transfers, LNS, and SBCC (Figure 1).

Furthermore, a formative research study will be conducted to design the SBCC package based on social cognitive theory and principles of social marketing using concepts from an integrated behavioral model. The implementation of the SBCC package will foster eating practices following the World Health Organization recommendation for PLW, adequate infant and young child feeding (IYCF) practices, use of LNS, and water, sanitation, and hygiene (WASH). A process evaluation will be conducted every 6 months to assess and identify bottlenecks, opportunities, and operational factors affecting the implementation. A cost analysis will also be conducted to assess the cost-effectiveness of each intervention.

### Ethics Approval and Consent to Participate

The Ethics Review Committee of Aga Khan University has granted approval for the proposed study (No. 4572-Ped-ERC-16). The National Bioethics Committee of Pakistan has granted approval for the study to be conducted on human participants (No. NBC-238). The trial is registered on Clinicaltrials.gov (NCT03299218). Written informed consent will be obtained from all study participants prior to enrollment in the study.

### Sample Size

A sample size was calculated to detect a 20% reduction in the prevalence of stunting among children aged 24 months, with 45% as the baseline prevalence of stunting among children aged 6 to 23 months [27]. The unit of randomization was the LHW’s catchment area, while the catchment area of 2 LHWs was considered one cluster. A sample size of 8 children per cluster was estimated with a statistical significance of *P*=.05, power of 0.80, and intracluster correlation of 0.0001. Each study arm has 50 clusters, from which 400 children aged 6 months will be enrolled in the study. A total of 2000 children will be enrolled in the study. The sample size was calculated using PASS software (version 11; NCSS LLC) [28].

BISP beneficiary households are defined as households with a poverty score of less than 16.17, based on indicators such as household size, housing structure, type of toilet facilities, education, child status, household assets, agricultural landholding, and livestock ownership. Children with severe acute malnutrition or chronic illness will not be enrolled and will be referred for treatment to the nearest public health facility.

For the formative research study and process evaluation, qualitative data will be collected through 80 in-depth interviews with key informants and 30 focus group discussions with mothers, fathers, mothers-in-law, and LHWs. Direct observations will also be conducted at household level to observe feeding practices and at community level to observe delivery of counseling sessions by LHWs every 6 months and at endline.

### Randomization

The unit of randomization will be catchment area of LHW. The randomization and allocation of study arms will be done by the data management unit (DMU) at AKU in Karachi, Pakistan using secondary data from BISP and the Punjab Health Department from the district Rahim Yar Khan. A total of 250 clusters, 50 in each study arm, will be randomized to the 4 interventions and 1 control arm.

### Eligibility Criteria

To be included in the study, households will have to meet 4 eligibility criteria: (1) BISP beneficiary for intervention arms and BISP poverty score between 16.18 and 20.00 for control arm; (2) living in the catchment area of LHW; (3) have at least one child aged 6 months at the time of inclusion; and (4) willing and able to provide written informed consent or thumbprint for the study.

### Data Collection

A total of 6 data collection teams will be hired locally from the district of Rahim Yar Khan for the study. Only women can collect data from mothers and their anthropometric measurements in the study area due to local cultural restrictions. Therefore, each team will consist of 2 female data collectors to interact with mothers and 1 male team leader to coordinate with fathers and male community members. The data collection teams will receive a 5-day hands-on training on study objectives, methods, data collection tools, techniques, anthropometric measurements, hemoglobin testing, and ethical issues. All questionnaires will be pretested in the field and changes will be incorporated accordingly before the actual data collection. A 1-day field test will be carried out before initiating field work. As part of the training, all field staff will be trained on anthropometric measurements with additional days for team measurers to continue to refine their skills. The training will include both in-class explanations and exercises with field practice. The training program will also include practice for weighing and measuring children.

Two trained staff will measure anthropometric measurements. The first measurer will measure and record each anthropometric measurement without revealing the values obtained to the second measurer. The second measurer will then independently repeat the same measurements. Each measurer will record their own values independently with no knowledge of the values recorded by the other measurer. After collecting the data, the 2 measurers will compare their measurements to ensure that the differences between their measurements fall within the standard maximum allowed differences (7 mm for length and 50 g for weight). Any pair of measurements that fall outside the maximum allowed differences will be repeated by both measurers and will be entered on the recording sheet. If this second pair of measurement values again exceeds the standard limits for that measurement, the measurers will repeat the measurement for a third and final time.

A structured questionnaire will be used to collect data on sociodemographic characteristics, parents’ and children’s anthropometric measurements, IYCF practices, immunization status, child morbidity, WASH, access and uptake of health services, exposure to other interventions, household food consumption, hunger scale, and household coping strategies at enrollment. The monthly follow-up questionnaire will be used to collect data on anthropometry (length and weight), compliance to intervention, morbidity, mortality, care-seeking patterns, and IYCF practices. Blood samples will be collected at 24 months of age for anemia and biochemical assessment.

Compliance data will be obtained by mother’s recall and comparing the number of used and unused sachets during each follow-up visit. Children’s weight will be assessed using a calibrated balance that allows double weighing (mother and child) and an automatic deduction of the mother’s weight to obtain the child’s weight. The scale (Seca GmbH) that will be used will have an accuracy of 50 g. Children’s length will be measured using a length measuring board (Seca GmbH) with an accuracy of 0.1 cm. Hemoglobin levels will be tested using HemoCue Hb 301 (HemoCue AB) analyzers.

A subset of households (5%) will be revisited for the purpose of data quality control and quality assurance. The entire process of data collection will be carried out on handheld tablets and will be supervised by team leaders and study managers, who will review data entry for errors and inconsistencies. The data will be stored in the MySQL Workbench (Oracle Corp) database at the DMU.

### Data Analysis

The primary independent variable will be the study arm, modeled as a 5-level categorical variable, with the control arm as the reference group. The primary outcome will be the prevalence of stunting, while wasting and underweight prevalence will be the secondary outcomes. Stunting will be defined as length-for-age *z* score (LAZ) less than –2 SD, wasting as weight-for-length *z* score (WLZ) less than –2 SD, and underweight as weight-for-age *z* score (WAZ) less than –2 SD. The primary outcomes will be measured at 6, 12, 18, and 24 months. Total number of monthly measurements with stunting, wasting, and underweight will be calculated by counting the number of times the child has a score less than –2 SD from WLZ, LAZ, or WAZ at a monthly measurement throughout the study period.

Since the study will be geographically clustered, prior to modeling, baseline characteristics will be compared by analyzing differences in means and proportions among the study arms using cluster-adjusted chi-square tests for proportions and analysis of variance for continuous variables. The adjusted analysis will account for the effect of baseline covariates found to differ across arms. A wealth quintile will be calculated using principal component analysis with data on household assets and characteristics.

For stunting, wasting, and underweight at individual time points (6, 12, 18, and 24 months), cluster-adjusted generalized linear models will be used with log link function to assess the risk ratios for each arm compared with control group. Negative binomial models will be built to examine incidence rate ratios for the total number of monthly measurements showing wasting, stunting, and underweight events. Gaps in measurement visits, and thus varying total number of months in which children were measured, will be controlled for using an offset of the natural log of the total number of months in which the child will be measured. All models will be checked for multicollinearity using variance inflation factors with cutoffs of ≥10 and assessed for influential outliers using leverage plots. All analyses will be performed with Stata statistical software (version 16; StataCorp). A cost-effectiveness analysis will also be conducted to quantify the net costs of the interventions and assess the costs per disability-adjusted life year saved.

## Results

The study protocol was approved by the Ethics Review Committee of Aga Khan University in Pakistan on January 4, 2017. Data collection began in May 2017 and was completed in July 2019. Data analyses are yet to be completed. This study will explore the effectiveness of intervention packages comprised of cash transfers from BISP with or without additional SNF, SBCC, or both in preventing childhood stunting. We expect the results to be published in peer-reviewed journals by autumn of 2020.

## Discussion

### Overview

The 2013 Lancet series on maternal and child nutrition estimated that a third of all child deaths attributed to malnutrition can be reduced by scaling up the provision of folic acid, multiple micronutrients, calcium, vitamin A, and balanced energy protein supplementation, along with adequate infant and child feeding practices for PLW and children in the most malnourished countries across the world [29]. To further capitalize on this, nutrition-sensitive actions are critical to eliminate undernutrition, as these actions address the lack of food security, adequate caregiving resources, access to health services, and a safe and hygienic environment, the key underlying determinants of maternal and child malnutrition [15]. Social protection programs are key, as they are inherently nutrition sensitive by targeting families at risk of malnutrition.

With the rising interest in and reach of social protection, there has been a rapid increase in countries with functional conditional cash transfer programs—64 countries in 2013 versus 2 in 1997. Similarly, an increased interest in the use of cash-based programs in vulnerable settings to prevent malnutrition has occurred, especially among governments [15].

There is considerable evidence on nutrition-sensitive interventions delivered through social protection programs addressing malnutrition in developing countries. These studies have mainly been conducted in Latin America and Africa, where institutional capacities within the health and food systems differ. In addition, local conditions and environmental differences make it difficult to generalize the results to South Asia [15].

The findings from this study will also improve understanding of which combination of interventions (ie, cash transfers, SBCC, and LNS) can create a greater impact on the reduction in the prevalence of stunting among children aged 6 to 23 months in a vulnerable setting. Furthermore, the findings from the cost-effectiveness analysis will improve understanding of which combination of interventions provides greater value to achieve the ideal nutrition objectives.

### Dissemination

The findings of this trial will be disseminated in a peer-reviewed journal to the governmental, academic, and policy-making communities, as well as to the wider public, and presented at relevant national and international conferences.

## Abbreviations

AKU: Aga Khan University
BISP: Benazir Income Support Programme
DMU: data management unit
IYCF: infant and young child feeding
LAZ: length-for-age z score
LHW: lady health worker
LMIC: low- and middle-income country
LNS: lipid-based nutrient supplement
PLW: pregnant and lactating women
SBCC: social and behavior change communication
UCT: unconditional cash transfer
WASH: water, sanitation, and hygiene
WAZ: weight-for-age z score
WLZ: weight-for-length z score
